# Addressing Younger Workers’ Needs: The Promoting U through Safety and Health (PUSH) Trial Outcomes

**DOI:** 10.3390/healthcare4030055

**Published:** 2016-08-10

**Authors:** Diane S. Rohlman, Megan Parish, Diane L. Elliot, Ginger Hanson, Nancy Perrin

**Affiliations:** 1 Oregon Institute of Occupational Health Sciences, Oregon Health & Science University, Portland, OR 97239, USA; diane-rohlman@uiowa.edu (D.S.R.); parish@ohsu.edu (M.P.); 2 Occupational and Environmental Health, University of Iowa, Iowa City, IA 52242, USA; 3 Health Promotion & Sports Medicine, Oregon Health & Science University, Portland, OR 97239, USA; 4 Center for Health Research, Kaiser Permanente, Portland, OR 97227, USA; Ginger.C.Hanson@kpchr.org (G.H.); Nancy.Perrin@kpchr.org (N.P.)

**Keywords:** young worker, eLearning, occupational, health protection, health promotion

## Abstract

Most younger workers, less than 25 years old, receive no training in worker safety. We report the feasibility and outcomes of a randomized controlled trial of an electronically delivered safety and health curriculum for younger workers entitled, PUSH (Promoting U through Safety and Health). All younger workers (14–24 years old) hired for summer work at a large parks and recreation organization were invited to participate in an evaluation of an online training and randomized into an intervention or control condition. Baseline and end-of-summer online instruments assessed acceptability, knowledge, and self-reported attitudes and behaviors. One-hundred and forty participants (mean age 17.9 years) completed the study. The innovative training was feasible and acceptable to participants and the organization. Durable increases in safety and health knowledge were achieved by intervention workers (*p* < 0.001, effect size (Cohen’s d) 0.4). However, self-reported safety and health attitudes did not improve with this one-time training. These results indicate the potential utility of online training for younger workers and underscore the limitations of a single training interaction to change behaviors. Interventions may need to be delivered over a longer period of time and/or include environmental components to effectively alter behavior.

## 1. Introduction

Approximately half of the United States’ 16 to 24-year-olds are employed, and that percentage increases by 10 percent during the summer months [1]. Younger workers experience twice the rate of occupational injuries as older workers [2]. Their heightened injury rate is influenced by many factors, including lack of job experience, not recognizing workplace hazards, and limited abilities to communicate effectively with supervisors [2,3,4,5,6]. In addition to work-related safety issues, developmental and lifestyle factors among this age group, such as binge alcohol use [7], balancing school and work demands [8], and sleep deficiency [9], may increase risks of an occupational injury.

Total Worker Health^®^ is a strategy that expands protection from work-related safety and health hazards to incorporate the promotion of health and wellbeing. Increasing evidence suggests that such a strategy may result in more efficient and effective achievement of both objectives [10,11]. In addition to their higher injury risk, an integrated approach may have unique benefits among younger workers. For example, adolescence is a time when healthy habits often decline, while harmful actions increase [12], and lifelong behavioral trajectories are initiated [13,14,15,16]. Thus, an approach addressing both occupational risk and more general lifestyle factors can potentially benefit these emerging adults throughout their lifetime.

Although addressing Total Worker Health^®^ among younger workers may have advantages, achieving this objective is a challenge. Despite employers being required to provide basic safety training for new employees, survey findings indicate that younger workers often received little or no training related to safety in the workplace [17]. A 2013 assessment found approximately one-third of younger workers reported no prior safety training [3], comparable to findings from almost a decade earlier [18].

Recognizing the needs of younger workers, the National Institute for Safety and Health (NIOSH) developed the Youth@Work: Talking Safety curriculum (www.cdc.gov/niosh/talkingsafety/). This six-part, 45-min-per-session teacher-led, classroom-based program is available on the NIOSH website. However, despite customized state-by-state resources, it has realized only limited reach and must compete against an already-overloaded high school curriculum [19].

Rather than a classroom-based curriculum, online technology offers a means to address the challenge of reaching younger workers. Almost all routinely interact online, most have smartphones, and more than three-quarters frequent social networking sites [20]. Adolescents and younger adults perceive the internet as a primary source for health-related information and prefer technology-enhanced educational programs [21]. Properly-designed online educational formats can be as effective as classroom programs for learning new material [22].

Promoting U through Safety and Health (PUSH) is a Total Worker Health^®^ intervention for younger workers, developed through the Oregon Healthy Workforce Center, a NIOSH Total Worker Health^®^ Center of Excellence. PUSH combines content from the Youth@Work: Talking Safety curriculum and two evidence-based adolescent health promotion curricula [23,24]. Content was modified and formatted for a computer-based instruction platform that has been effective in delivering occupational content for diverse worker groups [25,26,27]. The goal of the current study was to evaluate PUSH training feasibility, acceptability, and efficacy in a randomized, controlled trial among younger workers.

## 2. Methods

### 2.1. Study Population

All workers between the ages of 14 and 24 years old hired for summer employment by an urban parks and recreation bureau were eligible to participate. Most would work as lifeguards, swim instructors, or community center staff. When hired, all workers receive a basic safety orientation and are certified in CPR and first aid as a condition of their employment. Information about the study was presented at worker orientation during the summer of 2013; at this time, parental consent letters were also distributed to minors. Interested individuals provided their email address to research staff, and those potential participants were sent instructions for setting up user accounts on the study’s administrative website. The website allowed researchers to record consent, randomize participants, track their progress throughout the study, collect survey responses, link participant data across time points, and send individual and group emails (invitations, reminders, confirmations).

**Figure 1:**
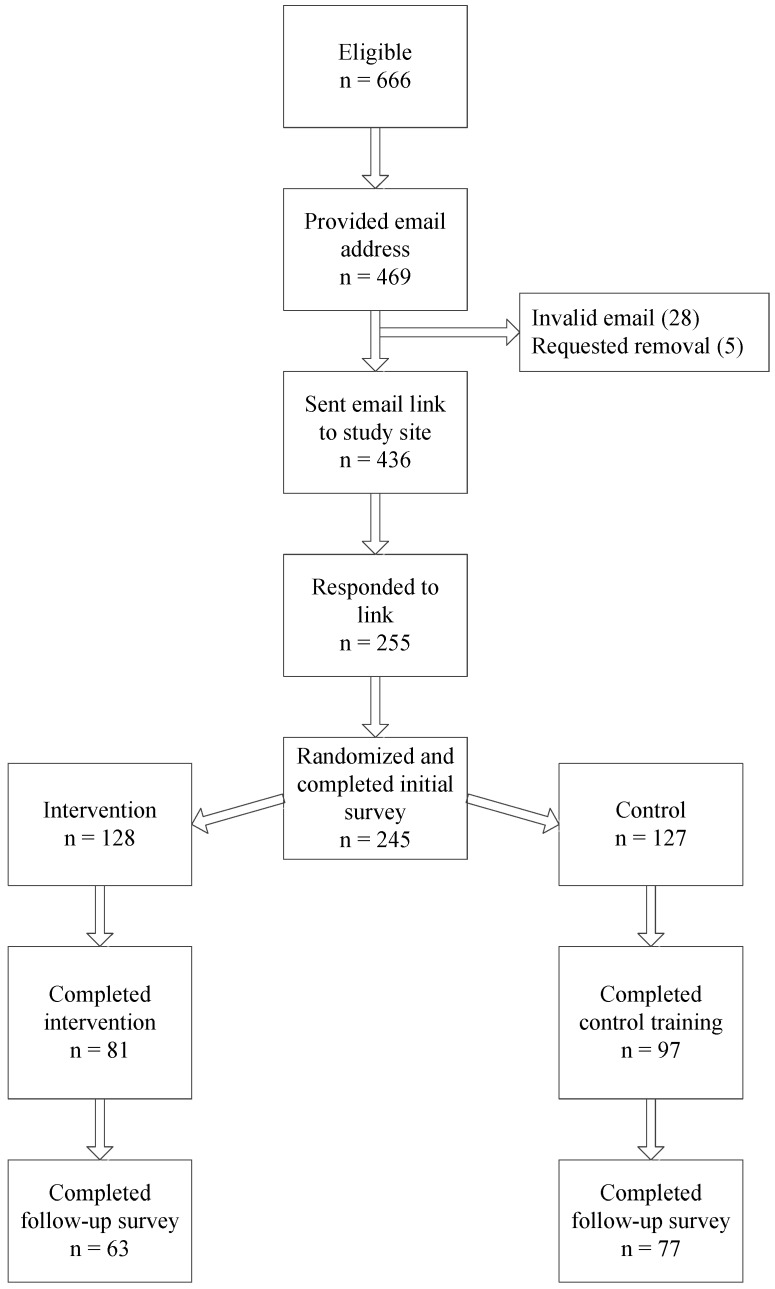
Participant recruitment and retention (data from those completing the intervention and control training were included in the analyses (*n* = 178)).

At the start of summer employment, those enrolled were sent a link to a confidential online survey. Following survey completion, an automated application on the study website used a random number algorithm to assign individual participants to the intervention or control condition and link them to the respective online training. At the end of their summer employment, six to eight weeks later, participants were sent a link to a follow-up survey also hosted on the administrative website. Participants were emailed a $15 gift card upon completion of the training and a $30 gift card after finishing the follow-up survey. Figure 1 shows the participation, randomization, and follow-up numbers.

Following orientation and study enrollment, employees were assigned to one job location, for example a neighborhood pool or community center. Accordingly, a given worksite had both intervention and control participants. Work supervisors were not aware of individuals’ group assignment. The Oregon Health and Science University Institutional Review Board approved all study materials and procedures.

### 2.2. Survey Instrument

At entry, participants provided demographic information, along with answering knowledge, attitude, and behavior items relating to occupational safety and lifestyle behaviors. The twenty-nine knowledge questions (Table 1) were content covered in the online training (safety, nutrition, physical activity, sleep, substance use, and communication skills), plus five additional items not included in the training. These multiple choice and true/false knowledge items were scored as either correct or incorrect. Participants’ responses were reduced to a percentage correct for the PUSH and non-PUSH items.

The attitude and behavior items related to safety and health made up four reliable constructs, which are shown in Table 2. For these items, participants responded using a seven-point agreement scale from strongly disagree to strongly agree. Thus, higher scores indicate more favorable responses. Construct scores were computed by taking the mean of the relevant items. At follow-up, intervention participants also were queried about reactions to the training and self-reported behavior change. The survey took 20 min, with the intervention and control training lasted approximately 1 h.

### 2.3. PUSH Training

**Figure 2:**
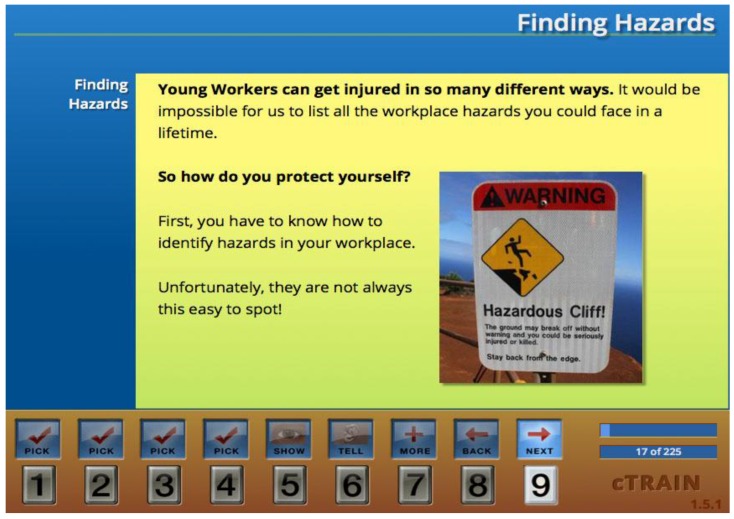
Example of the PUSH screen.

The PUSH training included topics derived from the NIOSH Youth@Work: Talking Safety curriculum, health promotion (nutrition, hydration, sleep, and substance abuse), and effective communication in the workplace. The online format used the cTrain platform [28], which is a format for self-paced computer-based training. An icon-based navigation system directed participants through a series of content screens, punctuated by brief videos. At intervals, multiple choice question screens reinforced content and required correct answers to progress through the training. Training was completed in approximately 60 min, and an example of a training screen is shown in Figure 2.

### 2.4. Control Condition

The control participants received a 50 min training using an established program on sun safety [29] and content about the benefits of positive thinking. The intervention participants did not receive this training.

### 2.5. Statistical Analyses

Data was analyzed with SPSS (IBM SPSS Statistics for Windows, Version 22.0, IBM Corp, Armonk, NY, USA). Chi-square and t-tests were used to examine differences between the groups at baseline. Generalized estimating equations (GEE) models were used to test differences in change from baseline to follow-up between the training and control groups’ attitudes and behaviors. GEE handles missing data by using the all available pairs method in which all non-missing pairs of data are used in estimating the working correlation parameters. Repeated waves of data are structured into a long dataset allowing for the use of all available data, so that cases are not lost due to missing data. Time, group, and time × group interaction were included in the model. Effects sizes were computed using the adjusted means and standard errors from the GEE model. Specifically, we computed the Cohen’s d as the difference in scores divided by the pooled baseline standard deviation.

## 3. Results

### 3.1. Participant Characteristics

Intervention and control participants’ baseline findings are presented in Table 3. Group demographics and body weights (body mass index (BMI) and percentages in BMI subgroups) were comparable at baseline, other than significantly more females being in the control condition. Gender was included as a control variable in the longitudinal analyses to statistically control for this baseline difference.

### 3.2. Feasibility and Acceptability

When queried at the end of their summer employment, the majority of intervention participants enjoyed the training (59%), reported that they learned new information (95%), self-reported that they had changed behaviors as a result of the training (63%), and would recommend it to others (67%). The parks and recreation department also found it easy to administer and requested making the training mandatory for all new employees the following year.

### 3.3. Program Effects

The pretest knowledge of participants was high (Table 1), and there were no differences between groups at baseline. Using GEE analyses to examine differences over time, while controlling for gender, the intervention participants had a significant increase in the number of correct PUSH knowledge items at the summer’s conclusion follow-up assessment. The adjusted means, standard error, effect sizes and *p*-value for each training × time interaction are presented in Table 4. The greater increase in knowledge in the intervention group was a small-moderate effect size (*d* = 0.40). Follow-up GEE analyses separating the PUSH knowledge items into three subscales: (1) communication, (2) health, and (3) safety, revealing that only the difference in change between intervention and control groups on safety knowledge was significant. Changes in the attitude and behavioral constructs also are shown in Table 4. Mean values decreased among intervention participants, although still remaining in the favorable range on the Likert agreement scale.

## 4. Discussion

PUSH is the first implementation of an online Total Worker Health^®^ training for younger workers. It integrated both occupational and non-occupational risk factors to more comprehensively address issues, and it used a format relevant for these technologically adept new younger employees. The training was easily administered and rated positively by participants. Results indicated that there were increases in knowledge among those completing the PUSH curriculum, and those favorable changes persisted during their summer employment.

PUSH used online training or e-learning, which broadly includes all forms of electronically-supported teaching [30]. A review of more than 20,000 worker education trials challenged the proliferation of employee educational videos and online trainings, noting that passively viewing a video, lecture, or viewing educational screens has limited ability to increase understanding and recall [31]. For example, less than 10 percent of lecture content is durably retained [32]. Those findings are well supported by adult learning theory’s emphasis on actively processing material for incorporating new knowledge [33]. However, unlike most trainings the cTrain platform differed in that it required mastery of knowledge items before progressing through the training, which forced participants to process material and facilitated the observed durable retention of PUSH knowledge content throughout the summer. The common wisdom is that knowledge-based programs have limited impact on behaviors, and longitudinal programs are needed to change actions [34,35,36,37]. We recognized that altering behavior with PUSH training would be a challenge, given its one-time nature and the constraints of having no follow-up worksite components to avoid diffusion of the intervention to control participants working at the same pool or other specific worksite. The findings underscore the limitations of both one-time training activities and the importance of including multiple levels of evaluation to assess the efficacy of worksite trainings. The American Society for Training and Development found that the majority of organizations only assessed training effectiveness with participant reactions [38]. Using that metric PUSH was rated positively by the majority of participants. A minority of employee trainings ask about whether behaviors were changed, as the assessments often immediately follow the video or online training. Using that “did you change” index, the majority of PUSH participants also reported changing their behaviors due to the training.

A more accurate measure of behavior is the pre-post-design, which is rare in most worker training programs. Using this rigorous index, PUSH did not find positive changes in attitudes or behaviors. Again, in this proof-of-concept study, without longitudinal and environmental components, behavioral changes were not expected. However, behaviors and attitudes appeared to decline, although still remaining at comparably favorable levels. What may explain the paradoxical findings relating to a decrease in attitudes and behaviors? A basic assumption of the pre- to post-test design is that the underlying metric used remains the same for both time points. However, as pre-test participants may have only a partial understanding of the items to be measured, at the end of the experience, their calibration may have changed. This response bias shift has been observed in other settings and poses a threat to the instrument’s internal validity [39,40]. As was seen here, participants tend to reduce their self-ratings after an intervention [38]. Rather than a negative finding, the decrease in values may represent heightened awareness of safety and health by PUSH intervention participants. This interpretation may be further supported by the participants’ generally positive reactions to the PUSH training, which demonstrated that youth enjoyed the training and self-reported that their knowledge and behavior had changed as a result of the training.

Our study has limitations. It was conducted in one geographic location, and participants, although representative of the community, were primarily white, well-educated and English speaking. Furthermore, many of the youth were employed as lifeguards, which is a safety-conscious occupation.

While knowledge may be permissive, it seldom alters behavior. Our findings emphasize the need for including reinforcing behavioral change with explicit messaging and worksite cues to safe and healthy actions. Enhancing PUSH with a follow-up system of text and social media messaging could reinforce and normalize safe and healthy actions. This sort of ongoing contact has been a component of online programs to alter younger adults’ diets and disease management behaviors [41,42,43] and could supplement the PUSH curriculum. In addition, including an environmental component, such as PUSH training-related worksite posters and supervisor reinforcement, would be predicted to reinforce attitude and behavior change [44].

## 5. Conclusions

Web-based training is a feasible and acceptable format for younger workers. As a stand-alone one-time interaction it produced sustained knowledge gains. Reinforcing content and adding environmental components may allow extending positive outcomes to attitudes and behaviors. The study also demonstrates the utility of pre- to post- assessments, rather than just self-reported change and reactions, when evaluating worker trainings.

## Figures and Tables

**Table 1:** Survey knowledge items and percentage correct at pre-test.

Knowledge Items	Pre-Test Percentage Correct
**PUSH Training Safety and Lifestyle Items**	
Best way to tell a coworker to stop horseplay	68.5
Which is a safety hazard	97.8
What to say if employer asks you to do something potentially hazardous	89.3
Which is not a biologic hazard	80.9
Best way to control a hazard	49.4
How to respond to being asked to work on new equipment without training	57.3
Best way to minimize effects of an emergency	90.5
When to wear appropriate personal protective equipment	88.8
How to respond to agitated customer	94.4
How to ask about your safety when asked to do a new task	83.7
How to talk with employer about safety hazard	83.2
What statement is not true about sexual harassment	79.8
Number of recommended servings of fruits and vegetables each day	47.2
Which is least healthy snack	53.7
How much exercise is recommended each day by the CDC	29.8
Which nutrient builds and repairs your body	93.8
Orange juice is what type of carbohydrate	57.9
You can be sleep deprived and not know it (T/F)	97.2
Chicken and fish are always the healthiest of the meat options at a fast food restaurant (T/F)	55.6
Alcohol and drug use by workers is related to more than half of all workplace injuries and fatalities (T/F)	76.4
If you are 16 years old and have a valid driver’s license you are allowed to drive a car on public streets as part of your job (T/F)	39.9
Practicing emergency protocols is an important part of preparing for emergencies (T/F)	98.3
The law says your employer is responsible for providing you with a safe and healthy workplace (T/F)	93.3
If you’re injured on the job, your employer must pay for your medical care (T/F)	62.9
**Non-PUSH Content Items**	
Which one is not an aspect of positive thinking	86.0
What is the first step to positive thinking	70.2
Skin cancer can be cured if it is caught early enough (T/F)	85.4
UV radiation is stronger around water, because the water reflects the sunlight (T/F)	91.0
Self-talk is the stream of unspoken thoughts in our head. Self-talk can be either positive or negative (T/F)	96.6

**Table 2:** Behavior and attitude constructs.

Construct	Items in the Construct	Alpha Reliability
Health Behavior	I bring healthy snacks to work	0.59
	I eat breakfast everyday	
	I stick to healthy food options when I eat out	
	I get at least 8 h of sleep a night	
	I sometimes drive when I am drowsy or tired *	
	I make time for exercise each day	
	I avoid engaging in behaviors before work that could jeopardize my attention and judgment	
Safety Behavior	I have looked at the emergency preparedness plans in my workplace	0.73
	I read the information about a chemical before I use it	
	I ask for help or training before trying a new task at work	
	I communicate professionally at work	
Health Attitudes	Hydration is important to staying focused and alert on the job	0.76
	I think eating breakfast everyday is important	
	Proper nutrition is important to workplace safety	
	Getting enough sleep at night is important to me	
	I know how to deal with my emotions in a healthy way	
	I think on the job injuries are a serious and common problem	
	An injury I receive on the job could potentially have a long-lasting negative impact on my life	
	I know how to protect myself from injuries in my workplace	
	I know how to identify hazards in my workplace	
	I have the ability to improve the safety of my workplace	
	I can make a difference in the safety of my workplace	
	I am confident I would respond appropriately	

* Reverse coded.

**Table 3:** Baseline descriptive information (percentages and means (standard errors)).

	Control (*n* = 97)	PUSH Intervention (*n* = 81)
**Demographics**		
Age (years) Age (years)	18.2 (0.2)	17.7 (0.2)
Percent Female Percent Female	60.8%	45.7% *
Percent White Percent White	77.1%	76.5%
Graduated High School	56.7%	45.7%
First Job	75.3%	81.5%
**Anthropometrics**		
BMI (self-reported height and weight)	23.2 (4.1)	22.7 (4.4)
Underweight Underweight	2.1%	2.5%
Normal Normal	77.9%	77.5%
Overweight Overweight	12.6%	12.5%
Obese Obese	7.4%	7.5%
**Attitudes and Behavior Constructs (1 to 7 scale, higher is healthier)**		
Health Behavior	4.9 (0.1)	4.9 (0.1)
Safety Behavior	5.5 (0.1)	5.7 (0.1)
Health Attitude	5.8 (0.1)	6.0 (0.1)
Safety Attitude	5.4 (0.1)	5.6 (0.2)
**Other Health Behaviors**		
Average Hours Sleep per Night	7.4 (0.1)	7.1 (0.1) *
Drink Until Drunk Past Month	26.8%	19.8%
**Diet ^‡^**		
Sugary Snacks	4.2 (0.2)	3.3 (0.2) *
Drinks with Added Sugar	3.6 (0.2)	2.8 (0.2) *
Fast Food	1.5 (0.1)	1.3 (0.1)
Meals from Home Fast Food	6.1 (0.2)	5.9 (0.2)
Fruits and Vegetables	6.6 (0.2)	6.3 (0.2)
**Exercise**		
At least 90 min each week	77.4%	85.2%

* Significant differences between control and intervention groups (*p* < 0.05); ^‡^ Diet items were answered using a frequency continuum from 0, never; 1 < once a month; 3, once or twice a week; 6, once a day; to 8, for three or four times a day.

**Table 4:** Scores for the knowledge plus safety and health behavior and attitude constructs (estimated mean (SEM)).

	Control *n* = 97		PUSH Intervention *n* = 81		*p* Value	Cohen’s d
	Pre-	Post-	Pre-	Post-		
**Knowledge Items**						
PUSH Training Knowledge Items (number correct)	18.2 (0.4)	18.2 (0.4)	18.4 (0.5)	20.2 (0.5)	<0.001	0.4
Non-PUSH Training Knowledge Items (number correct)	4.2 (0.1)	4.6 (0.1)	4.3 (0.2)	4.4 (0.2)	0.07	−0.21
**Safety and Health Behavior and Attitude Constructs**						
Health Behavior	4.9 (0.12)	5.0 (0.13)	4.9 (0.14)	4.9 (0.15)	0.220	−0.15
Safety Behavior	5.5 (0.15)	5.6 (0.15)	5.7 (0.17)	5.2 (0.18)	0.009	−0.36
Health Attitude	5.8 (0.13)	5.9 (0.13)	6.0 (0.15)	5.6 (0.16)	0.013	−0.37
Safety Attitude	5.4 (0.13)	5.6 (0.14)	5.6 (0.15)	5.4 (0.16)	0.015	−0.37
